# Redox-Dependent Inflammation in Islet Transplantation Rejection

**DOI:** 10.3389/fendo.2018.00175

**Published:** 2018-04-23

**Authors:** Jessie M. Barra, Hubert M. Tse

**Affiliations:** Department of Microbiology, Comprehensive Diabetes Center, University of Alabama at Birmingham, Birmingham, AL, United States

**Keywords:** redox signaling, reactive oxygen species, type 1 diabetes, islet transplantation, immune rejection, immunology, encapsulation

## Abstract

Type 1 diabetes is an autoimmune disease that results in the progressive destruction of insulin-producing pancreatic β-cells inside the islets of Langerhans. The loss of this vital population leaves patients with a lifelong dependency on exogenous insulin and puts them at risk for life-threatening complications. One method being investigated to help restore insulin independence in these patients is islet cell transplantation. However, challenges associated with transplant rejection and islet viability have prevented long-term β-cell function. Redox signaling and the production of reactive oxygen species (ROS) by recipient immune cells and transplanted islets themselves are key players in graft rejection. Therefore, dissipation of ROS generation is a viable intervention that can protect transplanted islets from immune-mediated destruction. Here, we will discuss the newly appreciated role of redox signaling and ROS synthesis during graft rejection as well as new strategies being tested for their efficacy in redox modulation during islet cell transplantation.

## Introduction

Type 1 diabetes (T1D) is an autoimmune disease characterized by chronic inflammation where self-reactive immune responses selectively target and destroy β-cells within the pancreas. In a majority of patients, insulin therapies can help regulate the rapid variations in blood glucose levels that result from this autoimmune attack, however, this is not a cure and for a relatively large number of patients, exogenous insulin treatment is not enough for them to maintain stable blood glucose levels (1). A major hurdle for insulin therapy is the ability to optimally sense and respond to glucose fluctuations as rapidly or precisely as a living β-cell. Therefore, the constant struggle to achieve efficient glucose homeostasis still persists. The death of these vital insulin-secreting cells within the islets of Langerhans and the resulting glucose dysregulation leaves patients at risk for developing serious life-threatening complications including cardiovascular disease, neuropathy, and renal failure (2).

Poor glucose control and the prevalence of secondary risks associated with T1D have lead researchers to investigate alternative treatment options for these patients. Unfortunately, in humans, there are currently no adequate metrics to detect these diabetic patients before the onset of autoimmunity. Usually, the presentation of symptoms, such as fatigue, extreme thirst, polyuria, or weight loss prompts a visit to a health professional, and only then does the presence of autoantibodies in their blood lead to their diagnosis. However, even at the time of diagnosis these patients still have some functioning β-cell mass remaining. Attempts have been made to delay or reverse the severity of T1D after diagnosis and to prevent further β-cell loss by utilizing immunotherapies to dampen autoreactive responses. A few of these therapies include the inhibitory cytotoxic T lymphocyte associated antigen-4 (CTLA-4)-Ig to block effector T cells (Teffs) (3), anti-CD20 therapy to deplete functional B cell responses and increase regulatory T cell (Treg) responses (4), and interleukin 1 beta (IL-1β)/IL-1R antagonists including anakinra and canakinumab to neutralize inflammatory signaling cascades including MAPK and NF-κB pathways (5). Clinical trials utilizing these treatments in early onset T1D patients displayed variable efficacy for maintaining higher C peptide levels with less reliance on exogenous insulin, however, any benefits were only temporary, and treatment was not successful in halting the progression of disease (3–5). The persistent challenges in early detection and the minimal effectiveness of immunotherapies have lead to a search for alternative treatment options to restore the functionality of insulin regulation in individuals after the destruction of β-cells has already occurred. One such attractive therapy is islet transplantation.

Islet cell transplantation is a more permanent alternative to exogenous insulin therapies with fewer long-term complications. By restoring functional β-cells into these patients, they can more accurately modulate their blood glucose levels and diminish the risks associated with glucose fluctuations. Unfortunately, as with any other organ or tissue transplant, immune-mediated graft rejection as well as an initial loss in islet graft viability induced by oxidative stress and inflammation continue to pose challenges for the long-term success of this strategy. There is also damage associated with the recurrence of autoimmunity toward the graft in the T1D patient as well as islet-specific risks of immunosuppression. In addition to the low survivability of the islet graft, other barriers to widespread utilization of this therapy include the sensitivity of islets toward hypoxia, redox-associated mechanical and chemical damage during isolation, and poor viability and islet yield from human cadaveric donors (6, 7). Subsequently, efficient human islet transplantation normally requires more than one cadaveric donor per recipient and some patients require consecutive transplants to prolong adequate blood glucose regulation (8).

Despite the hurdles that still need to be overcome, islet transplantation has come a long way in the last three decades. Prior to the late 1990s, islet transplantation into human patients had very little success in maintaining euglycemia and preventing hypoglycemic events, with few patients being insulin-independent beyond 1 week after transplantation (9). Since the 1980s, digestive enzymes and a mechanical shaking process known as the Ricordi method have been used to isolate islets (10, 11). Then, in 2000, a group led by Dr. James Shapiro at the University of Alberta published what would come to be known as the Edmonton protocol (12). This small clinical trial followed seven patients beyond 1 year after intraportal islet transplantation. The Edmonton protocol revolutionized the way human islets were isolated by utilizing xenoprotein-free isolation media and transplanting the purified islets directly after isolation, eliminating the dangers of islet culture. The islets were infused into the portal vein and utilized a novel combination of immunosuppression including sirolimus, low dose tacrolimus, and daclizumab, a monoclonal antibody that recognizes CD25. All recipients attained insulin independence for nearly 5 months after transplantation. This marked a profound improvement in patient outcomes compared to previous reports, and the protocol was soon adopted worldwide. In the nearly two decades since the Edmonton protocol was reported, advancements in our understanding of islet biology, islet graft viability, methods to protect islets following isolation, *in vitro* culture, and islet transplantation has improved. According to the 2016 Collaborative Islet Transplant Registry 9th Annual Report, 50% of recipients maintain insulin independence beyond 1 year posttransplantation, and around 20% of islet transplant recipients are insulin-independent after 5 years.

Ultimately, one challenge that still persists is the harmful side effects of immunosuppressive drugs to the patient as well as the islet graft (13). These anti-rejection medications inhibit the adaptive immune response; however, most of them do not protect the graft from redox-mediated destruction or direct autoimmune inflammatory interactions. In fact, the use of corticosteroids and tacrolimus can cause serious adverse effects including diabetogenicity and elevated extracellular reactive oxygen species (ROS) production in the islets themselves (14–17). It has been shown that immunosuppression with tacrolimus, sirolimus, and anti-IL-2R can even promote the proliferation of autoreactive memory T cells due to a chronic increase in serum IL-7 and IL-15 levels (18), potentially leading to a recurrence of autoimmunity. Tacrolimus and sirolimus have also been shown to impair mitochondrial calcium uptake and ATP production (19, 20), which are key steps in the glucose responsiveness of β-cells (21, 22).

Although the mechanisms that contribute to autoreactive immune responses in T1D and islet transplantation are not fully understood, what has become clear is the significant impact inflammation and oxidative stress can have on immune responses, β-cell function, and β-cell survival. Genetic attenuation of superoxide $\left({\text{O}}_{2}^{•-}\right)$ synthesis in the non-obese diabetic (NOD) mouse model through a point mutation in the nicotinamide adenine dinucleotide phosphate [NAD(P)H] oxidase (NOX) complex can influence innate and adaptive immune responses necessary for spontaneous diabetes progression (23–25). The inability to produce superoxide through the NOX complex highlights the important role of ROS generation and inflammation in disease progression, induction of β-cell death, and β-cell dysfunction (26). The generation of free radicals is not inherently a detrimental biological process, as ROS control apoptotic pathways within the cell, and the NOX complex is involved in eradicating microbial infections. While both of these responses are vital to cellular turnover and health, elevated ROS levels can influence cellular proliferation, survival, and the induction of inflammatory signaling cascades to mediate cellular damage (27). The dysregulation of ROS synthesis in an autoimmune setting can contribute to inappropriate activation of the immune system to recognize healthy tissue as foreign. This problem is particularly dangerous if an elevated level of ROS production overwhelms antioxidant defenses, which can result in oxidative stress, ROS-mediated damage, and eventual cell death (28).

In the context of islet transplantation, the role for redox signaling is even more vital due to the relatively low levels of native antioxidant defenses within the β-cell including superoxide dismutase (SOD), catalase, and glutathione peroxidase (Gpx-1), leaving them highly susceptible to ROS-mediated damage (6, 7). The impact of redox signaling within the context of islet destruction is twofold. The presence of oxidative species such as hydrogen peroxide (H_2_O_2_) and superoxide anions $\left({\text{O}}_{2}^{•-}\right)$ can impact glucose sensing within the β-cell (29), but they can also serve as a third signal to promote the maturation and expansion of β-cell-specific autoreactive T cell subsets (30–32). These autoreactive immune responses can initiate the destruction of β-cells though either the induction of apoptosis using the FAS pathway or by necrosis through the release of pro-inflammatory cytokines, perforin, granzyme B, and ROS (33, 34).

As scientists begin to appreciate the role of ROS in mediating inflammation and promoting transplant rejection, dissipating oxidative stress is a prime target for immunotherapies during islet cell transplantation to reduce islet vulnerability, boost patient outcomes, and prolong insulin independence (35). One proposed method to address these persistent challenges is to target the production of these reactive species during different stages of islet transplantation. The hope is that attenuating the redox status of the islets themselves or the surrounding microenvironment will promote islet function and prolong graft viability without the need for toxic immunosuppressive drugs.

## Immune Mechanisms Involved in Islet Transplantation Rejection

**Figure 1:**
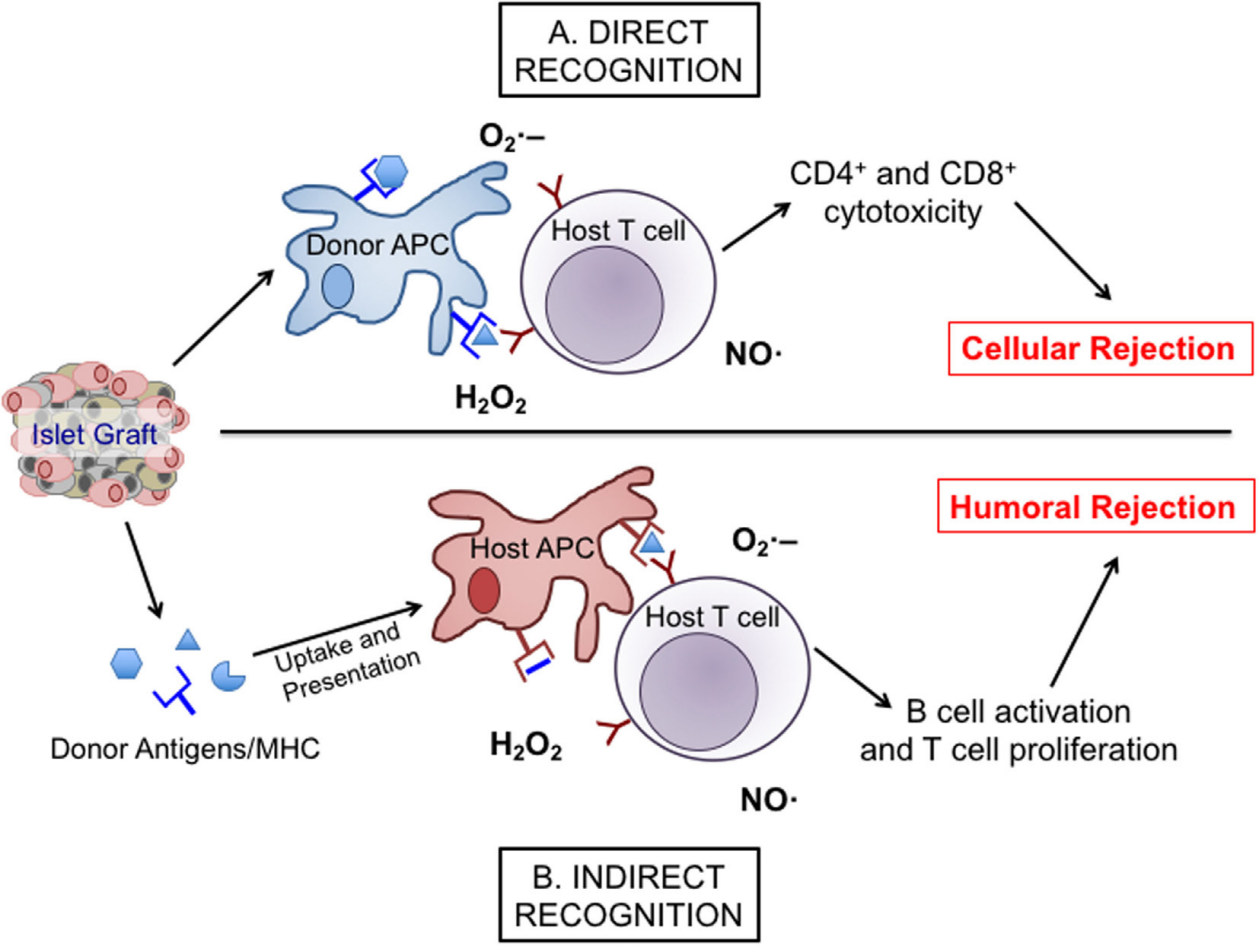
The direct and indirect recognition pathways of islet allograft destruction. After transplantation, islet-resident antigen-presenting cells (APCs) from the donor graft can present directly to host T cells with stimulatory signals provided by reactive oxygen species, such as ${\text{O}}_{2}^{•-}$, ^•^OH, and H_2_O_2_ to promote the expansion and activation of alloreactive cytotoxic T cell subsets. This stimulation can then activate inducible nitric oxide synthase (iNOS), NAD(P)H oxidase, and mitochondrial oxidative pathways within the T cell to produce more nitric oxide (NO), hydrogen peroxide, and superoxide, eventually leading to cellular rejection *via* the direct pathway **(A)**. Alternatively, islet antigens can be shed into the surrounding environment following transplantation to be engulfed and presented by host APCs and then indirectly presented to host T cells. Through co-stimulation and the release of ${\text{O}}_{2}^{•-}$, ^•^OH, H_2_O_2_, and NO, those APCs promote a classical antibody response involving the activation and expansion of alloreactive T cells and humoral rejection **(B)**.

Islet transplantation into patients with T1D comes with a risk for alloimmune responses as well as recurrent autoimmunity. Both responses can utilize redox signaling to facilitate their damaging effects on the islet graft. During allogeneic graft rejection, host immune responses can become activated through either direct or indirect recognition of donor tissues (Figure 1). Direct graft recognition involves the interaction of donor tissue-resident antigen-presenting cells (APCs) and host T cells through an MHC-mismatch interaction (36, 37). Indirect recognition involves the processing of donor graft peptides by host APCs to stimulate host T cells through the corresponding MHC interactions. Both direct and indirect recognition pathways require the involvement of co-stimulatory molecules to trigger and activate T cell responses. To understand why these aberrant signaling pathways and the corresponding redox responses are vital at various stages of islet transplantation, it is necessary to acknowledge the interplay between redox signaling and inflammatory responses. While others have examined certain specific pathways in great depth (38, 39), this review will highlight pathways involved in redox-dependent inflammation.

### Direct Recognition and Redox Signaling

In allogeneic transplantation, the direct recognition pathway involves donor APCs interacting with host effector CD4 and CD8 T cells to facilitate contact-mediated allograft rejection (40, 41). During islet transplantation, the direct recognition pathway stimulates a cellular rejection response in which direct killing of the islet graft by T cells is the primary endpoint (37). Several studies indicate that there are two requirements to execute direct islet allograft recognition and damage: the production of interferon gamma (IFN-γ) by T cells (42) and the initiation of apoptotic pathways through perforin and/or the use of Fas/FasL interactions between activated T cells and target tissues (43). Both of these mechanisms involved in islet allograft destruction have redox-dependent components that are intimately connected to their inflammatory responses.

The production of IFN-γ as well as other inflammatory mediators by APCs and by T cells is a tightly controlled process. One key regulator of inflammatory cytokine production involves the redox status of intracellular thiols. Reduced glutathione (GSH) is the most abundant free thiol in mammalian cells and is an important regulator of multiple cellular processes (44, 45). During stress conditions, GSH is oxidized into glutathione disulfide, leading to the activation of damage responses within the cell including the unfolded protein response (UPR) and apoptosis (45). Dendritic cells and macrophages with elevated levels of intracellular GSH produce more IFN-γ than those with low intracellular GSH levels (46, 47). This increased inflammatory profile by APCs can skew T cell responses through the synthesis of T cell polarizing cytokines such as IL-12 (47) and in an autocrine fashion to further promote APC activation (48). Once macrophages are activated, they produce large amounts of ROS as well as IFN-γ and IL-1β (38). These inflammatory signaling molecules aid in macrophage killing of target pathogens or facilitate islet graft destruction by mediating phagocytosis of β-cell debris.

Interferon gamma, tumor necrosis factor alpha (TNF-α), and IL-1β not only perpetuate damaging innate and adaptive immune responses but they also interact with their cognate cytokine receptors on the surface of the β-cell. Engagement of these β-cell surface receptors can initiate the activation of the RAS signaling cascade (49). Through a string of downstream phosphorylation events, the RAS pathway leads to the activation of MAPK and Myc, which can enter the nucleus and induce the transcription of genes involved in cell division, survival, and the production of inflammatory mediators (50). This pathway is redox-mediated through mitochondrial hydrogen peroxide activation of the Jun N-terminal protein kinase (JNK), which activates MAPK and stress pathways to further propagate inflammatory cytokine synthesis and apoptotic cell death (51).

The presence of ROS such as superoxide and hydrogen peroxide can also play a role during contact-dependent damage within the islet graft since the maturation of CD8 T cells to become cytolytic is a redox-dependent process (31). As a consequence of CD8 T cell activation by donor APCs, contact-mediated production of perforin and granzyme B by cytotoxic lymphocytes can permeabilize cells within the islet graft (52). Once these toxic molecules engage and enter the cell membrane, they initiate caspase-signaling cascades, which lead to either the direct initiation of cellular apoptosis through caspases-3 and -7 or to the cleavage of pro-apoptotic Bcl-2 family member, Bid, by caspase-8 (53). Bid then binds to the mitochondrial membrane and activates mitochondrial outer membrane permeabilization, stimulating the release of cytochrome *c* to kill the cell (54).

Redox regulation of apoptotic pathways has many facets that are detailed elsewhere (45), however, it is important to note that the primary role for ROS during apoptotic cascades involves intrinsic pathways. Mitochondria inside the β-cell can initiate stress-induced production of superoxide, which can then be converted into hydrogen peroxide and hydroxyl radicals (^•^OH) through the Fenton reaction (55). These radical ions are potent inducers of further redox-mediated DNA and protein damage inside the cell (56) and can lead to further apoptosis or inflammatory processes that negatively impact the viability of the graft.

### Indirect Recognition and Redox Signaling

Indirect recognition of allogeneic transplants involves the interaction between host APCs with host T cells. The host APC will process and present graft antigens on MHC II molecules to activate host CD4 T cells (40, 41). The indirect recognition pathway can promote two major forms of immune responses: the humoral B cell/antibody response through the interaction between CD4 follicular helper T cells with naïve B cells and the continual activation of innate responses, namely macrophage-associated killing (37).

The transition of a B cell to a terminally differentiated plasma cell requires various cellular and metabolic changes, some of which have redox components. H_2_O_2_ is involved in B cell receptor signaling and activation (57). In addition, as a B cell transitions to a differentiated plasma cell, the endoplasmic reticulum (ER) undergoes drastic reorganization and expansion. During ER stress, a host of ER-based enzymes generate ROS as byproducts, leading to multiple changes in antibody production, i.e., the switch to IgM (58) and proliferation within these newly formed plasma cells (59, 60). Specifically, it has been demonstrated that oxidation of Keap1, a negative regulator of the antioxidant response, allows for the nuclear internalization of Nrf2 and transcriptional activation of various target genes involved in B cell differentiation and antioxidant defenses (61).

After differentiation, antibodies produced by activated plasma cells can bind to the islet graft and activate the complement system to induce apoptosis within the target cell and facilitate islet graft destruction by cytotoxic lymphocytes through Fc binding (62). Antibody responses by B cells are not the only redox-dependent mechanism that can contribute to islet graft destruction. B cells are also capable of producing inflammatory cytokines including IL-6 that is redox regulated (63, 64). When a B cell receives co-stimulation through the CD40 surface receptor, the cross-linking of this receptor leads to the generation of ROS and subsequent activation of JNK pathways, resulting in the increased secretion of IL-6 into the surrounding environment (65, 66). IL-6 interacting with the cognate IL-6R can promote activation and proliferation of other immune cells by signaling through JAK2. This protein can initiate the MAPK cascade described above, or interact with STAT3, forming the JAK/STAT complex (67, 68). STAT3 is vital for optimal activation and effector function in T cells because it can directly enter the nucleus and initiate the transcription of inflammatory genes, or activate the NF-κB pathway and affect the cell cycle (69, 70). Therefore, redox regulation of B cell responses including antibody production and secretion of inflammatory cytokines can perpetuate damaging T cell responses to further destroy the islet graft.

## Role of Free Radicals and Pro-Inflammatory Mediators Involved in Islet Cell Transplantation Rejection

The interplay between free radicals and inflammatory molecules modulates β-cell dysfunction and death during multiple stages of purification from the pancreas and transplantation. Islets are sensitive to hypoxic stress or damage signals that occur during isolation and culture including pro-inflammatory cytokines and free radicals. Stress or damage caused by hypoxia, cold ischemia, and reperfusion can activate downstream inflammatory cascades including the NF-κB signaling pathway (71–73). After transplantation, immune effector cells including macrophages, neutrophils, B cells, and T cells migrate to the transplant site and target the islet graft for destruction by releasing pro-inflammatory cytokines, antibodies, and free radicals (74–76). Understanding the redox-dependent signaling pathways during islet isolation and following transplantation is vital to the development of novel interventions to improve transplantation success and prevent β-cell dysfunction.

### Redox Signaling in Islet Isolation and Culture

Pancreatic islets in their natural setting have rather high oxygen tension, with islets receiving more than 15% of the total pancreatic blood supply (77). This massive influx of blood and nutrients plays a key role in their rapid ability to regulate glucose levels, however, linked to their relatively low levels of antioxidant defenses, it also leaves them highly susceptible to ROS-mediated damage (6, 7). In addition, this sensitivity also further exacerbates their susceptibility to oxidative damage during isolation when they are deprived of that elevated oxygen supply, leaving them in a hypoxic state (78–80).

To separate islet cells from the surrounding tissue of the pancreas, harsh digestive enzymes and mechanical separation techniques are utilized to break down exocrine tissue while leaving the islets mostly intact. These methods, while efficient, induce a level of oxidative and mechanical stress as vascularization and in turn, the nutritional stores of the islets are severed (81). This detachment from the extracellular matrix leaves islets reliant on passive diffusion to survive the isolation and transplantation process (82, 83). Consecutive days incubated under hypoxic conditions *in vitro* can have serious impacts on islet function and survival. The increase in hypoxia and oxidative stress within *in vitro* cultured islets can induce DNA damage and the peroxidation of proteins and lipids (84, 85). Mitochondrial-derived stress can cause larger islets to develop a necrotic core as less oxygen is able to diffuse to the cells in the center (86) as well as impacting insulin secretion through stress-associated decreases in mitochondrial Ca^2+^ uptake (87). Islets compensate for the low availability of oxygen in culture by upregulating transcription factors including hypoxia-inducible factors, which induce transcription of multiple genes including toll-like receptors (TLRs) and genes involved in vascular endothelial growth factor (VEGF) signaling (88, 89). Hypoxic conditions can also activate NF-κB to induce the upregulation of inducible nitric oxide synthase (iNOS) and monocyte chemoattractant protein-1 (MCP-1) expression (71), which can have significant impacts on local inflammation after the islets are transplanted.

Strides have been made in recent decades in an attempt to combat oxidative stress with the advent of less damaging enzymatic digestion methods (90) and isolation techniques to improve cadaveric human islet yield (91), but challenges persist that motivate researchers to find alternative strategies to dampen oxidative stress and hypoxia. One method to protect islets from redox-mediated damage following isolation is to increase expression of detoxifying or antioxidant enzymes. Under normal circumstances, redox scavengers are upregulated in response to inflammatory signals released from cells during times of damage or stress. Unfortunately, the low levels of these scavenging enzymes in islets make it difficult for them to combat redox stress. A few of these key enzymes include SOD, manganese superoxide dismutase (MnSOD), and Gpx-1, antioxidant enzymes that are present at lower levels in islets than in other rodent tissues (7). Therefore, increasing endogenous antioxidant defenses or supplementing with exogenous scavengers could protect isolated islets from oxidative stress.

### Strategies to Dampen Redox-Mediated Damage in Isolated Islets

Mechanical and metabolic stress during islet isolation can significantly reduce the number of viable islets available for transplantation. To combat this early loss in islet mass, various groups have attempted to protect purified islets by targeting the redox mechanisms underlying sources of cellular stress. There are two primary techniques utilized to dampen oxidative stress and redox-mediated damage in purified islets: either supplementing culture media with exogenous redox scavengers like MnSOD or genetic manipulation of the islets themselves.

The first method to decrease the oxidative damage that islets endure in culture is to treat with antioxidants after isolation. The activation of both NF-κB and poly (ADP-ribose) polymerase pathways contribute to islet damage during the isolation process (92, 93). Dissipating oxidative stress through the use of a SOD mimetic can decrease NF-κB activation, reduce the production of inflammatory MCP-1 and IL-6 by human islet cells during stress conditions, and reduce ${\text{NO}}_{2}^{-}$ and ${\text{O}}_{2}^{•-}$ production by macrophages (93, 94). This same antioxidant demonstrated protection from STZ-induced apoptosis during *in vitro* human islet cultures as well as prolonged islet allotransplant survival in MHC-mismatched mouse models after purified islets were cultured in the presence of the SOD mimetic (95). Systemic administration of the SOD mimetic through the use of sustained release pellets prolonged the viability of allogeneic islet grafts by reducing immune migration to the site of transplantation. The reduction in inflammation and increase in graft viability observed in the above studies is a key step in protecting islets from oxidative stress produced by immune cells and can promote long-term survival of an islet graft. Similarly, a naturally occurring antioxidant from the extract of Chinese bayberries, cyanidin-3-*O*-glucoside (C3G), was shown to increase expression of heme oxygenase-1, Bcl-2, and survivin, antioxidant, and anti-apoptotic regulators that protect islet cells from oxidative stress *in vitro* (96, 97). In addition, C3G treatment of isolated islets prior to transplantation demonstrated prolonged graft survival with fewer islet numbers required to induce euglycemia when transplanted either under the kidney capsule or into the hepatic portal vein (97). The use of soluble antioxidants, while somewhat protective, is a short-term treatment option, and once these islets are transplanted, they are still susceptible to immune-mediated damage. Genetic modifications of purified islets may provide a more permanent solution and supply antioxidant protection that can persist long after transplantation.

Viral transduction of isolated islets to overexpress antioxidant genes provides benefits to islet survival not only during *in vitro* culture but also following transplantation. Transgenic mice overexpressing SOD and Gpx-1 within islets displayed a marked resistance to redox-mediated damage *in vitro* and improved glycemic control after transplantation under the renal capsule of syngeneic mouse recipients (98). One group genetically altered isolated murine islets to overexpress MnSOD and found that upon transplantation into immunodeficient recipients, the transgenic islets displayed a marked delay in graft failure following adoptive transfer with diabetogenic T cells (99). Similarly, transfection of islets with a lentiviral vector containing thioredoxin, an ROS scavenger, reduced the toxic effects of H_2_O_2_ *in vitro* and prolonged graft viability after transplantation into the kidney capsule of spontaneously diabetic NOD mice (100). Other groups have also shown protective effects of glutamylcysteine ligase and SOD overexpression on islet function (101, 102), further supporting the important role of antioxidant defenses and oxidative stress for the maintenance of islet graft function.

These studies have focused on the treatment of islets after isolation, however, in human islet isolation, another hurdle also exists. Most human islets isolated for transplantation are obtained from cadaveric or brain dead organ donors. Unfortunately, these donor conditions create an elevation of inflammatory and redox-mediated damage to human tissues that can negatively impact islet yield. Therefore, it is not surprising that human islet transplant recipients can require three or more donors to obtain sufficient islet equivalents for a single transplant (103). If human islets will continue to be used for transplantation, the state of the donor before isolation cannot be ignored. One group found that treatment of brain dead rats with exendin-4, a glucagon-like peptide-1 analog that acts to increase insulin secretion and decrease glucagon production (104), served to prevent islet viability loss induced by brain death-related inflammation as well as increasing glucose-stimulated insulin secretion of these isolated islets (105). In addition, exendin-4 has also been shown to reduce hypoxia-related islet injury, reduce oxidative stress, and improved both syngeneic and xenotransplantation survival in mouse transplants (106).

While dissipation of oxidative stress during isolation and culture is important to improve islet yield, viability, and function from donors for transplantation, there are numerous other redox-dependent insults transplanted islets have to withstand to delay graft failure including immune-mediated inflammation. Therefore, defining how the two arms of the immune system facilitate islet transplant rejection, graft failure, and synergize with oxidative stress is important to prolong the survival of transplanted islets into patients with T1D.

### Acute Responses and Redox Signaling After Islet Transplantation

Following transplantation, islets are susceptible to acute mechanisms of stress that lead to the loss of a large portion of islet cell mass and function (107). One such stress includes ischemia reperfusion injury as these islet cells are placed back into living tissue. The rapid influx of a multitude of nutrients as well as soluble factors not seen in culture media induces an inflammatory response involving oxidative stress known as the instant blood-mediated inflammatory reaction (IBMIR). This reaction is a nonspecific response by the innate immune system that causes robust coagulation and immune infiltration into and around the islets (88, 108), which leads to the induction of cellular apoptotic signaling pathways and internal activation of oxidative stress within the β-cell.

During allogeneic transplantation into the hepatic portal vein, IBMIR-associated responses cause an instantaneous activation of complement pathways that can lead to thrombosis and significant loss of the islet graft (109, 110). One of the major initiating factors in this response is the expression of tissue factor (TF) by islet endothelial cells (ECs). This factor can lead to the activation of thrombin, platelet activation, and secretion of other inflammatory mediators that can perpetuate inflammation and induce macrophage-directed killing (111–113). Once this cascade has begun, upward of 60% of the islet graft is lost within the first few days (82, 107). This local inflammatory cascade at the site of transplantation can induce tissue-resident macrophages to produce superoxide and hydrogen peroxide to damage surrounding tissues (114, 115). The outflow of these reactive molecules can directly lead to DNA strand breakage and peroxidation of proteins or lipids while also activating a number of signaling pathways shown to induce apoptosis in the vulnerable islet graft (116). Not only can local redox signaling originating from the site of transplantation during IBMIR impact islet survival but also the functionality and glucose responsiveness of the β-cells. Therefore, targeting this reaction immediately after islet transplantation is a good technique to prevent the early loss of islet mass.

Another key innate immune mechanism of inflammation during this early stage of islet transplantation is the release of danger signals known as danger-associated molecular patterns into the extracellular space. These danger signals are highly pro-inflammatory, being recognized by pattern recognition receptors (PRRs) on innate immune cells as well as by epithelial cells (117). One key subset of PRRs are TLRs, which recognize specific pathogen-associated molecular patterns, including lipopolysaccharide, dsRNA, flagellin, and unmethylated CpG. Signaling through these TLRs can lead to downstream activation of MyD88, a myeloid differentiation adaptor protein that plays a key role in signal transduction associated with the activation of immune responses (118, 119). Once activated, MyD88 can initiate NF-κB-dependent transcription, one of the major transcription factors involved in the inflammatory response toward the islet graft (120). One group found that inhibition of MyD88 dimerization with small molecule TJ-M2010-6 in NOD mice displayed reduced onset of diabetes, inhibited insulitis, and suppressed T cell activation (121).

### Targeting Redox-Mediated Acute Responses After Islet Transplantation

There are multiple ways to target these acute interactions and prolong graft survival. One of the major stages of IBMIR is coagulation and platelet aggregation around the islet graft. To diminish this damaging reaction, one group utilized α-1 antitrypsin, a serine protease inhibitor to reduce IBMIR coagulation and cytokine-induced inflammation in human islets transplanted into the portal vein of NOD.*scid* mice (122). Administration of α-1 antitrypsin reduced TF expression by the islets, inhibited neutrophil infiltration, and protected islet grafts from IBMIR-mediated damage. Another strategy involves developmental endothelial locus-1 (Del-1), an endothelial-derived homeostatic factor that has anti-inflammatory properties due to its involvement in leukocyte adhesion (123, 124). The overexpression of Del-1 reduced leukocyte-platelet aggregation, which protected islets from IBMIR-associated damage (124). Alternatively, targeting the production of TF by the islets themselves can be inhibited by nicotinamide treatment (113).

The damage from IBMIR can cause an outflow of cytokines from these early immune effectors that can activate redox-sensitive signaling cascades within the islets. These redox-dependent pathways induce apoptosis within the islet graft and compromise insulin secretion from β-cells (125). In human islets, it has been shown that IFN-α can participate in the early stages of T1D progression by triggering ER stress responses to reduce insulin production (126). This change in insulin production was linked to a functional delay in the rate of proinsulin to insulin conversion within the ER. The role of oxidative stress in this ER response has also been investigated. In particular, it has been suggested that the production of iNOS and nitric oxide (NO) within isolated islets after cytokine exposure can lead to the activation of UPR within the ER (127, 128). During times of cellular stress, misfolded proteins can accumulate within the ER lumen. When this build up occurs, it can cause damage to cellular functions as well as disrupt cell division and survival (129). The UPR cascade is designed to protect the cell by increasing protein degradation, upregulating transcription of protein folding machinery, and reestablishing proper ER function. However, if the UPR is incapable of compensating for the amount of cellular stress, such as in the case of chronic inflammation following islet transplantation, the cell can undergo apoptosis. This has been shown to occur during spontaneous T1D progression in the NOD mouse model. ER stress responses were demonstrated to precede the onset of hyperglycemia in the NOD mouse, establishing a link between redox signaling, ER stress, and the early wave of islet dysfunction seen in T1D models (130). Pre-diabetic NOD mice displayed β-cells with fewer secretory granules and a more fragmented ER when compared to β cells from diabetes resistant mouse strains.

It has been shown that UPR defects in β-cells from both animal models of T1D as well as from human patients can contribute to the pathogenesis of autoimmune diabetes (131). Blocking UPR hyperactivation through the use of tyrosine kinase inhibitors such as KIRA8 and imatinib displayed reductions in ER stress-induced apoptosis and even reversed autoimmune diabetes in the NOD mouse (132, 133). In addition to autoimmune diabetes, targeting the UPR during ER stress may be a potential therapeutic target to delay islet transplant rejection. Negi et al. provided evidence of ER stress being implicated in the high degree of human islet loss during isolation and during the early posttransplantation period when these islets were engrafted into a chronic hyperglycemic environment (134). To circumvent ER stress and apoptosis, one group found that pre-treatment of human islets with glial cell line-derived neurotrophic factor reduced ER stress and improved graft function after transplantation into the kidney capsule of diabetic immunodeficient mice (132, 133, 135).

In addition to ER stress, one of the major problems during islet transplantation is the early loss of functional insulin-producing cells due to hypoxia-related injury (107, 136). Redox reactions are tightly linked to hypoxic and reoxygenation conditions as the cellular electron transport chain of the mitochondria become damaged (136, 137). Due to the intimate link between mitochondrial function and insulin secretion, the mitochondrial stress induced by hypoxic or reoxygenation conditions can induce β-cell dysfunction. It has been demonstrated that even transient exposure to H_2_O_2_ can reduce β-cell glucose responsiveness by upward of 40% long after the stress has been removed (138). This decrease in responsiveness correlated with increased mitochondrial ROS and decreased mitochondrial biogenesis, solidifying the link between internal sources of oxidative stress and β-cell dysfunction. In addition, dissipating mitochondrial ROS through antioxidants such as MitoTempo or Mitoquinone can protect β-cells from oxidative damage and increase insulin responsiveness in diabetic conditions (139). Not only can mitochondrial ROS impact insulin secretion but it can also induce further DNA and protein damage (55). Therefore, potentially reducing mitochondrial ROS-mediated damage through the use of redox regulators could protect islets from this initial loss of functional β-cell mass in the few weeks after implantation (85).

To address this problem of reoxygenation, researchers have attempted to use gene delivery or co-culture methods to promote revascularization of these islets in the days after transplantation, thereby limiting oxidative damage and loss of early graft function. One popular method to achieve this is the use of VEGF. This particular growth factor gained attention due to its limited ability to cause secondary side effects as compared to cytokines like transforming growth factor-β (140). Using an adenovirus-based delivery system, the induction of elevated levels of VEGF in human islets resulted in a protective effect from TNF-α and IFN-γ induced apoptosis (141). Researchers also found that the addition of VEGF promoted revascularization in human islets transplanted under the kidney capsule of mouse recipients by promoting the growth of new blood vessel formation (142). Co-expression of VEGF and an IL-1R agonist demonstrated suppressive effects on cytokine- and consequently redox-mediated necrosis and apoptosis (143). Therefore, combinatorial therapies including VEGF expression and IL-1β-dependent signaling blockade demonstrate promise in maintaining stable islet engraftment and function.

Transplantation studies targeting these early immune responses demonstrate some protection for the islet graft; however, the islet graft is still susceptible to immune-mediated damage from adaptive immune effectors. Therefore, targeting one pathway may not be sufficient to prevent redox-mediated islet destruction. Dissipation of only one subset of free radicals provided a modest protective effect and negligible improvement in islet function (144, 145). Perhaps a more comprehensive blockade of redox signaling mechanisms during islet transplantation would improve the duration of islet viability. Support for this hypothesis is demonstrated through the use of cell-permeable catalytic antioxidants, which are effective in delaying streptozocin-induced islet cell death, and decreasing the synthesis of inflammatory cytokines and free radical production by immune cells (31, 94, 95). Treatment of rat islets with metallothionein, a broad antioxidant involved in a wide array of protective stress responses (146), can restore and maintain euglycemia after subcutaneous islet transplantation, a result not seen in untreated control transplants due to the challenge of revascularization under the skin (147). An even more drastic effect was seen with the use of fusion proteins combining metallothionein and SOD to target multiple sources of free radical damage, leading to improved graft survival of syngeneic transplantation models in mice (148).

**Figure 2:**
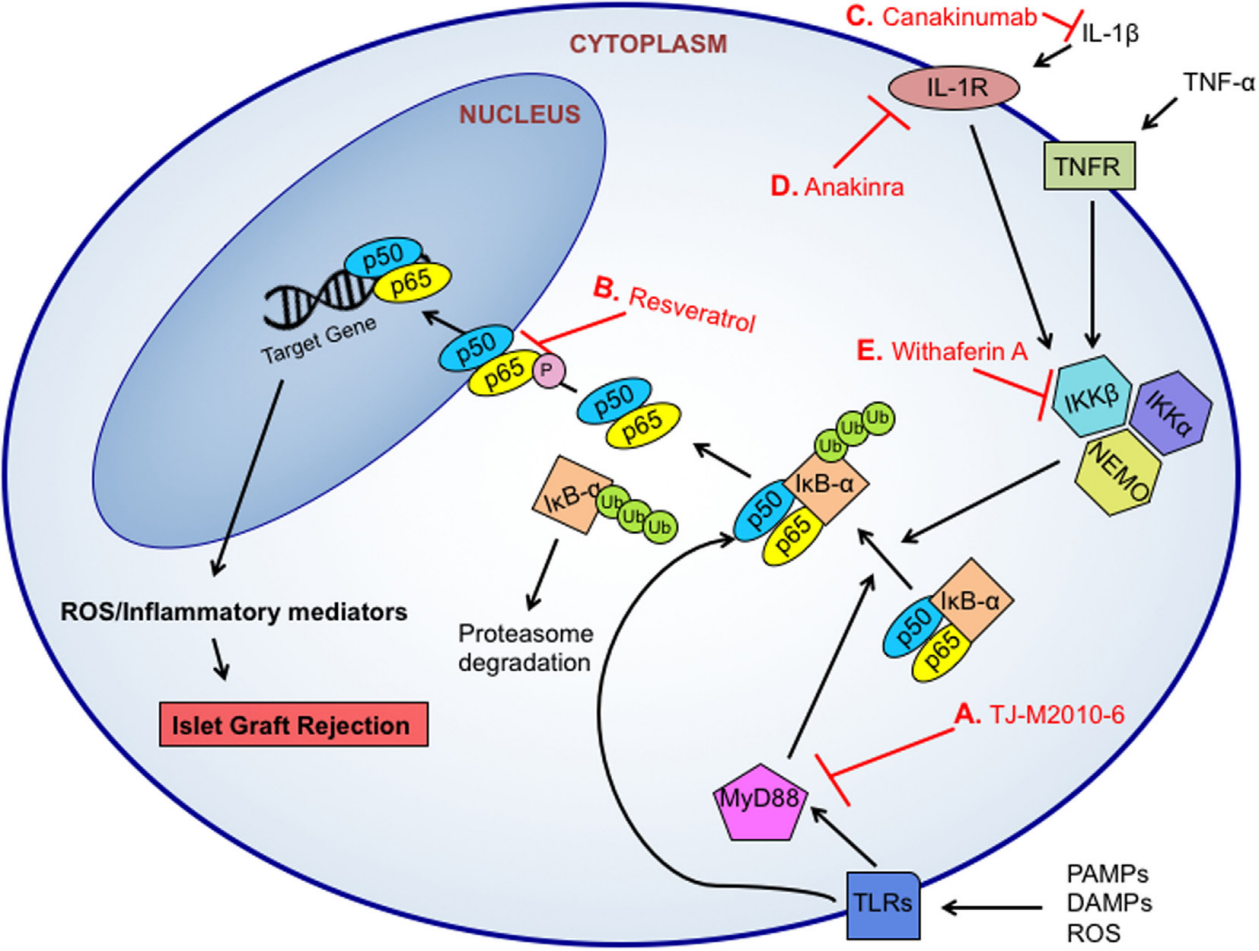
Targeted therapeutic approaches for NF-κB inhibition. NF-κB can induce the transcription of various inflammatory and oxidative molecules to facilitate islet graft rejection. The NF-κB pathway can be triggered by pro-inflammatory cytokine signaling, pathogen-associated molecular patterns (PAMPs)-, danger associated molecular patterns (DAMPs)-, or reactive oxygen species (ROS)-initiated toll-like receptor (TLR)-dependent signals. TLR-signaling activates the MyD88-dependent or MyD88-independent signaling pathways, which results in IKK phosphorylation, IκB-α degradation in the proteasome, and NF-κB (p50/p65) nuclear translocation. Small molecule inhibitors like TJ-M2010-6 can prevent MyD88 activation and IκB-α degradation **(A)**. Resveratrol inhibits the phosphorylation and subsequent nuclear localization of the NF-κB p65 subunit to prevent transcription **(B)**. Signaling from cytokines like interleukin 1 beta (IL-1β) have been targeted through the use of monoclonal antibodies including anti-IL-1 canakinumab **(C)**, which blocks binding of IL-1β to its receptor, and the use of IL-1R antagonist anakinra to block signaling through the receptor **(D)**. The IKK complex is a target for NF-κB inhibition. Specifically, inhibition of IKKβ and NEMO complex formation by withaferin A can prevent the phosphorylation and release of IκB-α **(E)**.

Taken together, redox-meditated destruction of islet cell grafts can be initiated and perpetuated by a multitude of different signaling pathways induced by immune cells and β-cells. With its multiple intersections with inflammatory responses as well as the production of redox molecules such as an increase in iNOS expression and production of ${\text{O}}_{2}^{•-}$, the NF-κB pathway has become a major target for therapeutic intervention (Figure 2). Using two common NF-κB inhibitors, withaferin A, which inhibits IKKβ and NEMO complex formation (149), or an analog of resveratrol, which blocks the phosphorylation and subsequent nuclear localization of the p65 NF-κB subunit (150), Kanak et al. found that NF-κB blockade reduced the release of C-peptide and proinsulin as well as the production of pro-inflammatory cytokines and chemokines including TNF-α, MCP-1, IL-8, and IL-6 in *in vitro* human islet and blood co-cultures (88). The use of a natural NF-κB inhibitor, withaferin A, is another good example. The addition of withaferin A induced a decreased expression of five key inflammatory genes, *RANTES* (CCL5), *IP10* (CXCL10), *MIG* (CXCL9), *IL1B*, and *NOS2* when islets were cultured in the presence of a cytokine cocktail as compared to controls (151), indicating a strong anti-inflammatory response in addition to a reduction in redox mediators involved in this vital signaling cascade.

While the blockade of this vital pathway has shown some potential at reducing inflammatory responses, understanding the functional and redox-dependent mechanisms involved in activating these pathways at various stages of islet transplantation is critical to understanding the immune-mediated pathology of islet cell destruction and graft failure. With these overlapping mechanisms in mind, it is not surprising that targeting a single pathway may not be efficacious in eliminating the challenges facing the field of islet cell transplantation. One example is the use of imatinib, which hinders the non-receptor tyrosine kinase c-Abl. This drug was initially used to treat chronic myeloid leukemia; however, several clinical trials also demonstrated improvement or reversal of diabetes phenotypes (152, 153). In animal models, imatinib demonstrated protection from both spontaneous and drug-induced islet death and dysfunction (154–156), and when investigated further, imatinib treatment of human islets demonstrated a decrease in islet inflammation following cytokine exposure (157). There has also been some data indicating that imatinib treatment may be capable of reversing autoimmune diabetes in NOD mice by blunting the ER stress responses within pancreatic β-cells (132, 133). With these biological roles, researchers believed imatinib would be a potent inhibitor of redox-mediated apoptotic pathways. However, when used for *in vivo* transplantation studies, pre-treatment of islet cells or treatment of recipients posttransplantation did not improve islet transplant outcomes (158). The failure of imatinib treatment to protect islet grafts *in vivo* serves as a reminder that oxidative damage and redox signaling is complex and a potent mediator of multiple pathways involved in graft failure.

While the above therapies are promising techniques for the reduction of inflammatory reactions, transplanting antigenic islets and delaying graft rejection into a recipient with established autoimmune diabetes is a herculean task. Not only will there be issues of MHC incompatibility in these allotransplant settings, which will potentially mark the graft for destruction, but also an inherent autoimmune response primed and ready to produce signaling molecules and oxidative species to immediately attack the transplanted β-cells is also present. The development of novel therapies that can efficiently decrease adaptive immune responses involved in graft destruction is necessary if there is hope for diminishing graft rejection without the use of immunosuppressants.

### Therapies Targeting Adaptive Immune Rejection of Islet Grafts

There are multiple strategies being investigated to suppress the adaptive immune responses that contribute to islet graft destruction, however, two in particular have gained more attention in the last few decades: islet encapsulation strategies to provide a barrier between the sensitive islet graft and the immune system and co-transplantation methods using accessory cells to dampen inflammatory immune responses. Both seek to provide immuoprotection to the islet graft while maintaining the ability for the β-cells to respond to environmental stimuli.

Current strategies for protecting the islet graft against adaptive immune rejection utilize inhibitors of some of the most commonly utilized pathways for inflammatory responses.

One method to provide protection from immune-mediated damage without perpetual dependency is encapsulation of isolated islets with materials designed to delay immune rejection. These materials have gained attention for their potential to provide an immunoprotective and physical barrier between the immune system and newly transplanted islets. Encapsulation aims to produce a semi-permeable membrane around islet cells that allows insulin and other nutrients access across the membrane while excluding larger proteins like antibodies or interactions with immune cells (159). There are three common methods used for encapsulation: (1) an intravascular device, (2) macroencapsulation, and (3) microencapsulation (160–162).

The first category requires the use of a small chamber containing multiple islets, that is, then directly connected to a blood supply (160), and while this type of intravascular device was capable of restoring euglycemia in mouse models (163), the threat of thrombosis made this method unreasonable for clinical use. Macroencapsulation of islets does not require direct attachment to a blood supply and is more attractive for clinical application. However, the thickness of these capsules can impede the transfer of insulin, oxygen, and other nutrients, potentially harming the islets and limiting possible transplantation sites (164, 165). More recently, the development of new technologies including the subcutaneous implantation of islets held within a thin membrane-bound device by TheraCyte can protect insulin-producing cells from the immune system and delay islet allograft rejection (166, 167). In addition, a device by ViaCyte utilizing PEC-01 precursor insulin-producing cells and a subcutaneous transplantation site is currently in a phase 1 clinical trial. Finally, microencapsulation is the encapsulation of a single islet, attempting to address the porosity and mass issues that plagued the earlier methods. Reducing the width and the number of encapsulated islets improves porosity and reduces redox-related injury, however, retrieval of transplanted islets is more difficult (161, 168). Finally, alginate is typically used for islet micro- and macroencapsulation, but is also innately immunogenic due to an inability to generate a completely pure form of this algae-derived compound (169, 170).

While each method above has shown some success in restoring euglycemia in animal models and clinical trials (171), the inability to consistently control the size, shape, and thickness of these capsules continue to hamper long-term success. In addition, addressing the issue of reactive species, which may be small enough to cross these semi-permeable membranes, continues to pose a challenge and is a source of much debate (168, 172). In an attempt to address the setbacks associated with these encapsulation materials, the congregation of microspheres containing suppressive materials with or around these islets may provide a solution. Microspheres are specialized structures comprised from thin layers of cross-linked polymers, which can then be optimized for porosity to suit the desired cellular effect (173, 174). Because this method relies less on a bulky shell, microspheres offer the flexibility to address larger issues such as the cellular microenvironment, both within and outside the capsule. For example, the congregation of curcumin, an anti-apoptotic drug containing free radical scavenging capabilities, with the polymer poly(lactic-co-glycolic acid) to form heterospheroids can decrease oxidative stress and bolster insulin release in rat islets when used as an encapsulation material (174). This technique allows for a localized release of the redox-modulating drug directly at the site of transplantation without degradation in circulation.

Another novel method of redox-dependent protection that does not compromise size, permeability, or charge of the islets is the use of a layer-by-layer (LbL) polymer ultrathin coating. These nanothin layers allow for the manipulation of surface area, permeability, and bioreactivity of the encapsulation material while still providing protection to the encapsulated islets (175). This technique allows for the potential aggregation of different materials into a single, confluent capsule and opens the door for addressing multiple mechanisms of islet transplant destruction. Furthermore, in contrast to other methods, the LbL technique does not require a priming step for adherence of the biomaterial to the islet surface, which has been shown to be detrimental to the stability and viability of islet cells (176). Instead, these ultrathin coatings rely on hydrogen bonding between the lipid polymer and the lipid bilayer of the membrane to form an anchor point which binds the polymer to the surface of the cell (177).

Utilizing this LbL technique, capsules composed of poly(*N*-vinylpyrrolidone) and tannic acid (TA), a powerful antioxidant, can suppress the production of IFN-γ and TNF-α, pro-inflammatory cytokines, which are key players in islet cell destruction (178, 179). These capsules can scavenge ROS as well as reactive nitrogen species produced by immune cells, demonstrating their redox-modulation capacity. The TA-containing capsules are also efficacious in suppressing pro-inflammatory chemokine production by innate cells, leading to a decrease in T cell trafficking to the site of inflammation and a decrease in T cell activation (180). By reducing immune cell trafficking, these capsules not only serve as a physical barrier to immune destruction but also serve in a localized manner to suppress immune responses without eliciting global immunosuppression.

Other strategies to reduce early loss of β-cell mass include co-transplantation with accessory cells that can enhance islet function, prevent apoptosis, promote vascularization, and provide immunoprotection, including mesenchymal stem cells (MSCs), ECs, Tregs, and myeloid-derived suppressor cells (MDSCs) (181). MSCs are mesodermal multipotent cells that have self-renewing properties and can be isolated from almost every adult tissue (182). They can surround purified islets in culture due to their strong adhesive capabilities and improve islet graft viability and revascularization in both rodent and non-human primate models of co-transplantation (183–185). MSCs can suppress inflammatory immune responses including the proliferation of cytotoxic T cell subsets in part through NO synthesis and inhibition of STAT5 phosphorylation (186). In islet transplantation, Mohammadi Ayenehdeh et al. demonstrated that congregation of adipose tissue-derived MSCs with isolated islets in a hydrogel could maintain euglycemia for more than 30 days during intraperitoneal allotransplant (187). This prolonged islet survival was in part due to an increase in Treg populations as well as a reduction in the inflammatory cytokines IFN-γ and IL-17A.

Another population being investigated for its resistance to IBMIR reactions is ECs. Co-culture of human ECs with isolated pig islets was shown to prevent IBMIR-mediated islet damage both *in vitro* (188) as well as after co-transplantation into the kidney capsule of diabetic immunodeficient mice (189). Another group found that co-culturing isolated human islets with primary human ECs produced a protective coating that would surround the islets and protect them from IBMIR upon transplantation into the portal vein (190). This co-transplantation strategy also induced a reduction in CD11b^+^ innate immune cell infiltration into these islet grafts, indicating that the presence of ECs served as an immunoprotective barrier for transplanted islets.

An innate immune cell type that has gained attention for its contact-dependent immunosuppression is the MDSCs. Through their production of superoxide, iNOS, and elevated arginase activity, MDSCs can suppress Teff activation and function while promoting Treg development (191, 192). During allogeneic islet transplantation into the kidney capsule of diabetic mice, co-transplantation of MDSCs increased the presence of Treg cells through the B7-H1/PD-1 pathway (192) and reduced CD8 T cell infiltration by activating iNOS (193).

Regulatory T cells are an immune cell population with the potential to prevent islet graft rejection due to their suppressive effects on immune responses. A recent clinical trial demonstrated that *ex vivo* expansion and subsequent infusion of autologous human Treg cells in 12 patients with newly diagnosed T1D lowered the patients’ exogenous insulin requirements and prolonged endogenous islet survival (194). The use of Treg cells also show some promise in animal models of islet transplantation, however, due to the short half-life of expanded Tregs as well as challenges in migration from peripheral blood to the site of engraftment (195), alternative strategies to improve Treg localization have been attempted. The combination of CTLA-4, a key protein receptor that downregulates immune responses, and reparixin, which blocks against inflammatory neutrophil infiltration, resulted in lower serum IFN-γ as well as decreased T cell infiltration into the islet graft after transplantation under the kidney capsule (196). Other groups have investigated the use of fusion proteins and antibodies to promote graft survival. Zhang et al. utilized CTLA-4/Fc and demonstrated reduced local inflammation, a concomitant increase in Foxp3^+^ Treg cells, and improved engraftment (197). Treg cells have also been utilized in co-transplantation strategies where co-aggregation of syngeneic Treg cells with purified allogeneic islets within an agarose hydrogel displayed prolonged allograft survival after transplantation into the portal vein of mice (198).

## Conclusion

**Figure 3:**
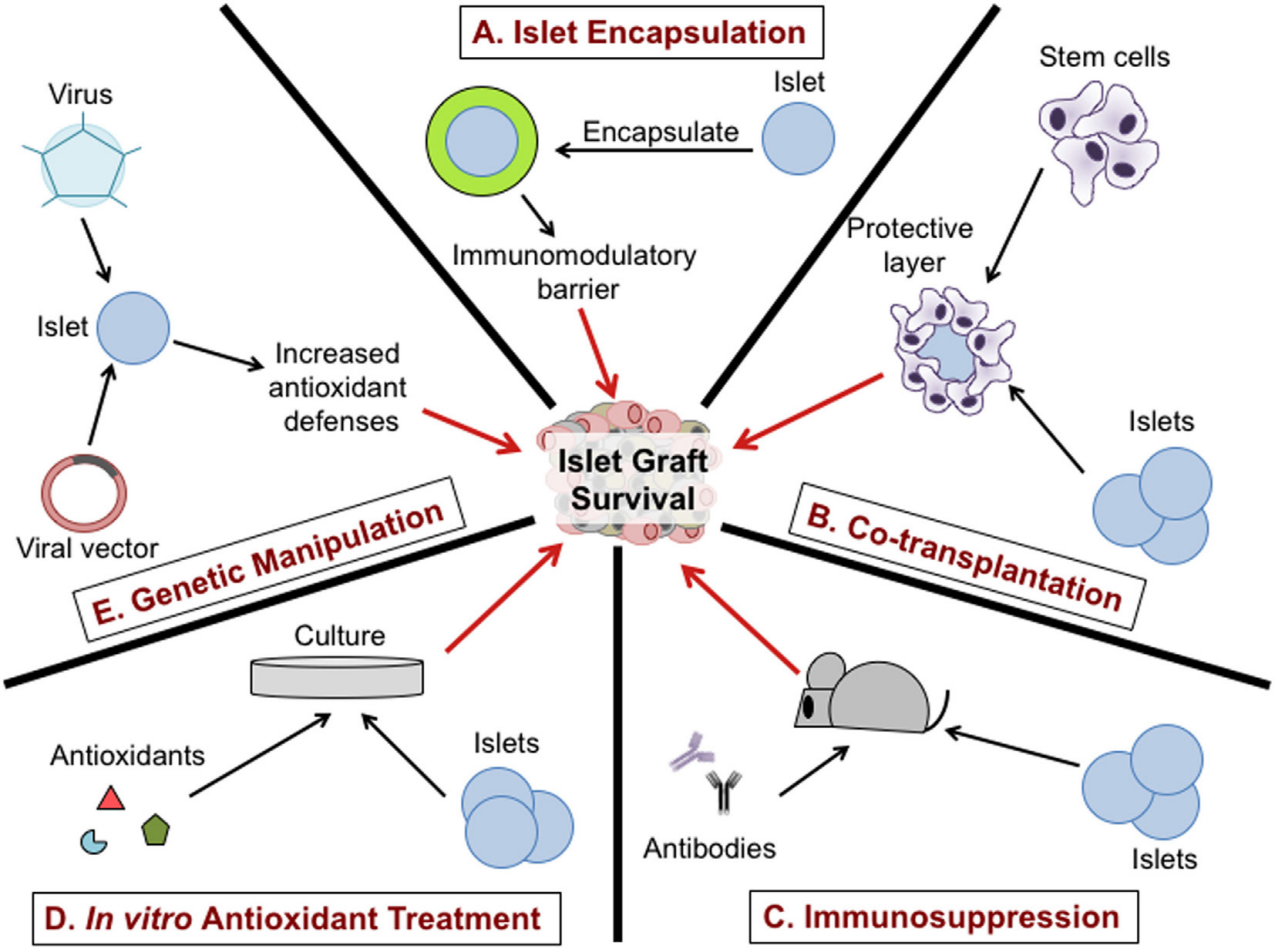
Therapeutic approaches to protect islet graft viability. Encapsulation of purified islets provides a physical and potentially immunomodulatory barrier between the islet graft and the host immune system **(A)**. Co-transplantation of stem cells or regulatory immune cells with the islet graft can reduce immune infiltration to the graft **(B)**. The use of immunosuppressive drugs such as monoclonal antibodies specific for immune cell subsets can suppress inflammatory responses in the host and prolong graft survival **(C)**. Treatment of purified islets with antioxidants during culture can dissipate free radicals involved in islet dysfunction and viability, may enhance engraftment and delay graft rejection **(D)**. Transfection of purified islets using a virus or viral vector to increase antioxidant defenses can promote islet graft viability and prolong survival after transplantation **(E)**.

Due to the comprehensive role of oxidative stress on islet transplantation, targeting redox-dependent inflammatory responses during islet isolation, *in vitro* culture, and after transplantation has the potential to increase islet viability and function (Figure 3). Utilization of a broad range of antioxidants including SOD mimetics to dissipate ROS synthesis can prolong islet viability and maintain function during islet isolation (93). The addition of a physical barrier as well as an immune modulator is likely the most promising combinatorial approach to protect islet transplants from immune-mediated rejection. Interventions including islet encapsulation with TA may be efficacious and safer in delaying allograft rejection than global immunosuppressive therapies (12, 15, 162, 180). In an effort to achieve stable islet engraftment in patients following islet transplantation, therapies that specifically target the removal of free radicals and redox-dependent signaling are highly warranted. It is apparent that synergistic interactions between redox biology and immune responses following islet transplantation are an underrepresented area of research. Future strategies implementing the LbL encapsulation approach in combination with potent antioxidants can enhance the viability and yield of isolated islets, making islet cell transplantation a realistic and curative treatment option for patients with T1D.

## Author Contributions

JB and HT wrote the manuscript and reviewed/edited manuscript. HT is the guarantor of this review article.

## Conflict of Interest Statement

The authors declare that the research was conducted in the absence of any commercial or financial relationships that could be construed as a potential conflict of interest.

## Abbreviations

APC, antigen-presenting cell; CITR, Collaborative Islet Transplant Registry; CTLA-4, cytotoxic T lymphocyte associated antigen-4; DAMPs, danger-associated molecular patterns; DCs, dendritic cells; Del-1, developmental endothelial locus-1; ECs, endothelial cells; ER, endoplasmic reticulum; Gpx-1, glutathione peroxidase; GSH, glutathione; GSSG, glutathione disulfide; HIF, hypoxia-inducible factor; HO-1, heme oxygenase-1; H_2_O_2_, hydrogen peroxide; IBMIR, instant blood-mediated inflammatory reaction; IFN-γ, interferon gamma; IL-1β, interleukin 1 beta; iNOS, inducible nitric oxide synthase; JNK, Jun N-terminal protein kinase; LbL, layer-by-layer; MCP-1, monocyte chemoattractant protein-1; MDSCs, myeloid-derived suppressor cells; MnSOD, manganese superoxide dismutase; MOMP, mitochondrial outer membrane permeabilization; MSCs, mesenchymal stem cells; NAD(P)H, nicotinamide adenine dinucleotide phosphate; NOD, non-obese diabetic; NOX, NAD(P)H oxidase; ${\text{O}}_{2}^{•-}$, superoxide; ^•^OH, hydroxyl radical; PAMPs, pathogen-associated molecular patterns; PARP, poly (ADP-ribose) polymerase; PRRs, pattern recognition receptors; PVPON, poly(*N*-vinylpyrrolidone); RNS, reactive nitrogen species; ROS, reactive oxygen species; SOD, superoxide dismutase; TA, tannic acid; Teff, effector T cell; TF, tissue factor; TGF-β, transforming growth factor-β; TNF-α, tumor necrosis factor alpha; Treg, regulatory T cell; T1D, type 1 diabetes; UPR, unfolded protein response; VEGF, vascular endothelial growth factor.
